# Study of EGFR mutations in head and neck squamous cell carcinomas

**DOI:** 10.4322/acr.2021.251

**Published:** 2021-04-19

**Authors:** Gurpreet Kaur, Deepika Phogat, Venkatesan Manu

**Affiliations:** 1 INHS ASVINI, Department of Pathology, Colaba, Mumbai, India; 2 151 Base Hospital, Department of Pathology, Guwahati, Assam, India

**Keywords:** Biomarkers, Neoplasms, Genes, erbB-1, Genomics

## Abstract

**Introduction:**

Squamous carcinoma is the commonest malignancy of the head and neck region. It is associated with high morbidity and mortality. Epidermal growth factor receptor (EGFR) regulates downstream signaling pathways through its tyrosine kinase (TK) domains that play a role in cell proliferation and survival. EGFR mutations have been found to occur between exons 18 to 21 on chromosome 7. Limited studies are available on EGFR-TK mutations in the head and neck squamous cell carcinoma (HNSCC) globally. This study explores EGFR mutations in 30 HNSCC cases presenting to a tertiary care hospital over a period of two years.

**Material and Methods:**

Fresh tumor tissue was collected from the resection specimens of cases of primary HNSCC. Cases with pre-operative therapy were not included. Parameters in the form of patients’ age, gender, smoking/tobacco intake, site of the lesion were recorded. Tumor parameters after histopathological examination were recorded in the form of TNM stage, tumor grade. DNA was extracted from fresh tissue of all the cases. EGFR Mutation Analysis Kit assay was used to detect mutations of the *EGFR* gene. PCR was run and results were analyzed.

**Results:**

*EGFR* Mutations were found in 6.7%of the patients. There was no significant association of the *EGFR* Mutation with the studied parameters.

**Conclusion:**

*EGFR* mutations are present in a subset of patients of HNSCC. Patients having these mutations may benefit from targeted therapy with tyrosine kinase inhibitors.

## INTRODUCTION

Squamous cell carcinoma is the most common malignancy of the head and neck region.^1^ It is associated with high morbidity, and mortality.^2^ Recent genomic studies showed a molecular heterogeneity associated with this entity, which involves multiple carcinogenesis pathways.^3^ Various risk factors play a role in developing head and neck squamous cell carcinoma (HNSCC) like tobacco, alcohol, and virus such as human papillomavirus (HPV).^4^ ^,^ ^5^ Self-sufficiency of growth signals is one of the hallmarks of carcinogenesis. The epidermal growth factor receptor (*EGFR),* a receptor for the epidermal growth factor (EGF), is composed of transmembrane and cytoplasmic tyrosine kinase domain, which controls downstream signaling pathways that play a role in the cell division and survival. Downstream signaling pathways like Mitogen-Activated Protein Kinase (MAPK), phosphatidylinositol-3- kinase (PI3K)-AKT, and Signal Transducer and Activator of Transcription (STAT) are responsible for various steps in carcinogenesis like cell division, cell migration, and angiogenesis.^6^ The *EGFR* Tyrosine Kinase (TK) domain mutations have been found to occur on chromosome 7 from the exon 18 to 21. These comprise of a single base deletion, insertions, or substitutions. *EGFR* mutations have been studied in solid organ tumors like non-small cell lung carcinoma. Patients having these mutations have shown good response to targeted therapy with EGFR-tyrosine kinase inhibitors (TKIs) like Gefitinib.^7^ ^,^ ^8^

Studies on *EGFR* mutations in cases of HNSCC are limited. Therefore, a study of the presence of mutations in *EGFR-TK* in our population may improve therapies for these patients. This study explores the presence of mutations in the *EGFR-TK* gene on chromosome 7 between exons 18 to 21 in 30 HNSCC patients presenting to a tertiary care hospital for two years.

## MATERIAL AND METHODS

A cross-sectional descriptive study was done to study *EGFR* mutations in squamous cell carcinoma of the head and neck (HNSCC), to evaluate *EGFR* mutations in cases of HNSCC and correlate *EGFR* mutations with the various patient and tumor parameters in a tertiary care hospital.

Institutional ethics committee clearance was obtained prior to proceeding with the study. The study was carried out on all cases of biopsy-proven squamous cell carcinomas of the head and neck, presenting to a tertiary care hospital over two years.

All consecutive cases of Head and Neck region diagnosed as squamous cell carcinoma (HNSCC), and in which resection specimens of the primary tumor was received at the Department of Pathology, tertiary care hospital, starting from Jan 2016 up to Dec 2017 were compiled.

The cases that received, preoperatively, radiotherapy, and/or chemotherapy were excluded from the study. Secondary metastatic tumors to the head and neck were not included.

The minimum number of patients in our study to achieve statistical significance was calculated and found out to be 30.

Fresh HNSCC tissue was collected from the resection specimens of 33 cases (Jan 2016- Dec 2017). The tissue was stored in saline at -40 Celsius degrees. Parameters in the form of patients’ age, gender, smoking/tobacco intake, duration of symptoms, and the lesion site were recorded. After histopathological examination, tumor parameters were recorded in the form of the TNM stage as per AJCC 7th edition, Grade of the tumor as per Broders’s criteria into well, moderately, and poorly-differentiated, lymphovascular invasion, perineural invasion, and extra-nodal tumor extension.

DNA was extracted from fresh tissue of all the cases and the quality was assessed by nanodrop spectrometry. Three samples had to be rejected due to the suboptimal quality of DNA. Mutational analysis was done on the remaining 30 samples.

Genomic DNA was isolated using the Pure Link Genomic DNA Kit by the kit protocol and quantified using the nanodrop.

DNA concentration of greater than 10 ng/microliter and A 260/280 ratio greater than 1.8 was considered adequate. (A 260/280 ratio less than 1.8 indicated more protein content). This assay required approximately 80 ng of DNA for each sample per run (10 ng/reaction). Lower concentrations were adjusted with the water volume during the reaction setup.

The *EGFR* Mutation Analysis Kit (*EGFR* Mutation Analysis Kit Instructions for use For In Vitro Diagnostic Use EntroGen, Inc. 7) assay was used to detect mutations in exons 18, 19, 20, and 21 of the *EGFR*.

The mutations detected in each reaction were:

- p.T790M (Exon 20);
- Exon 19 Deletions - detects 19 deletions, but did not distinguish between them;
- p.L858R (Exon 21);
- p.L861Q (Exon 21);
- p.S768I (Exon 20);
- p.G719* (Exon 18) - detects G719A, G719S, and G719C, but did not distinguish between them;
- Exon 20 Insertions - detects c.2319_2320 ins CAC and c.2310-2311 ins GGT, but did not distinguish between them;
- Exon 20 Insertion - detects c. 2307_2308 ins9 GCCAGCGTG.

The endogenous control primers were run to amplify an unrelated gene used to determine the condition of reagents and whether the reaction contains a sufficient amount of amplifiable DNA.

The regents were prepared as per the kit instructions, and the PCR reactions were set up in a total volume of 30microlitre/sample.

The PCR was run on Roche Light Cycler R 480 (Software version 1.5), and results were analyzed.

## RESULTS

**Figure 1:**
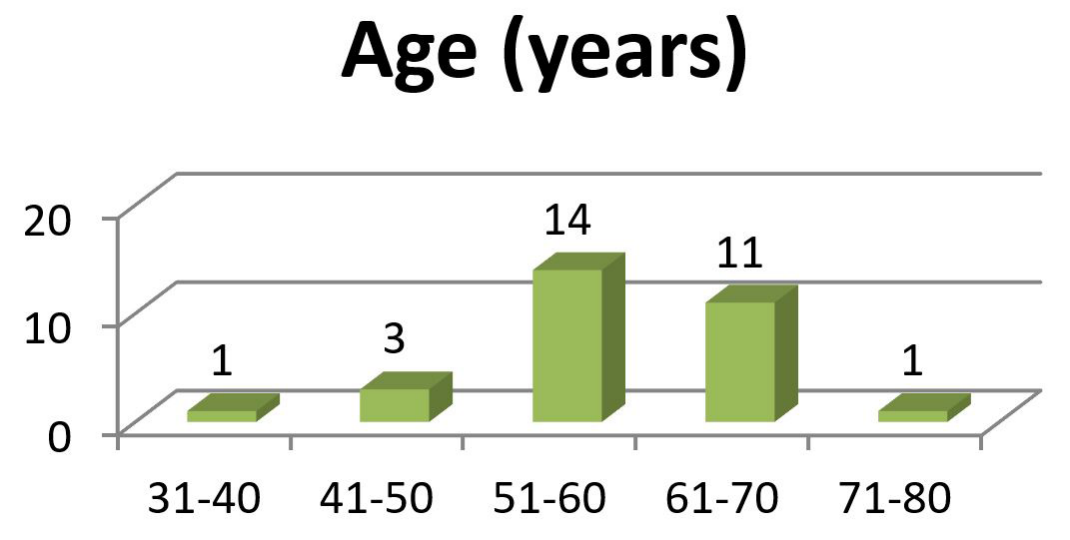
Distribution of patients according to Age

The mean age of the patients was 58.2 years (Figure 1). There was a male preponderance (83.3%).

The most common site of the tumor was the tongue (33.3%), followed by the buccal mucosa (20%), mandible (10%), and maxilla (6.7%). One case was found on the floor of the mouth, gingivobuccal sulcus, glottis, larynx, lip, oropharynx, supra-glottis, tonsillar fossa, and tonsillar pillar.

50% of the patients chewed tobacco, while 26.7% were smokers. The TNM stage was (i) T1 in 11 cases (36.7%), (ii) T2 in X cases (13.3%), (iii) T3 in 3 (10%) patients, and (iv) T4 in 11 (36.7%) patients. The majority of the patients (76.7%) had Grade 1 tumors, while 5 (16.7%) and 2 (6.7%) patients had Grade 2 and Grade 3 tumors, respectively.

In our sample, 1 (3.3%) patient presented lymphovascular invasion, perineural invasion (PNI) was found in 1 (3.3%) patient, and extranodal extension was observed in 2 (6.7%) patients.

*EGFR* mutations were found in 2 (6.7%) patients. One patient had a single mutation (p.S768I), while the other patient had two mutations (p.S768I, p.T790M).

It was observed that both the *EGFR* mutations were located in the exon 20.

Both the patients with *EGFR* mutation were in the age group of 51-60 years. Both the *EGFR* mutations were positive in the tumor of the tongue as the primary site.

Data were analyzed using appropriate statistical tests in unpaired t-test, Fisher test, student t-test, and chi-square test.

There was no significant association between *EGFR* mutations with age, gender, or history of tobacco intake. There was no significant association of *EGFR* mutation and stage or grade of the tumor (Tables 1 [Table t02] [Table t03] [Table t04]-5).

**Table 1:** Association of EGFR Mutation and Age of patients

Age	EGFR Mutation				Total		p Value
	Present		Absent				
	N	%	N	%	N	%	
31-40	0	-	1	3.3%	1	3.3%	>0.05
41-50	0	-	3	10%	3	10%	
51-60	2	6.7%	12	40%	14	46.7%	
61-70	0	-	11	36.7%	11	36.7%	
71-80	0	-	1	3.3%	1	3.3%	
Total	2	6.7%	28	93.3%	30	100%	
Mean ± SD	53.5 ± 2.12		58.6 ± 8.57		58.2 ± 8.38		

N= Number of patients having EGFR mutation. SD= Standard Deviation. p Value= Probability Value.

**Table 2 t02:** Association of EGFR Mutation and Gender of patients

Gender	EGFR Mutation				Total		p Value
	Present		Absent				
	N	%	N	%	N	%	
Male	1	3.3%	4	13.3%	5	16.7%	>0.05
Female	1	3.3%	24	80%	25	83.3%	
Total	2	6.7%	28	93.3%	30	100%	

N= Number of patients having EGFR mutation. SD= Standard Deviation. p Value= Probability Value.

**Table 3 t03:** Association of EGFR Mutation and Tobacco intake

Tobacco intake	EGFR Mutation				Total		p Value
	Present		Absent				
	N	%	N	%	N	%	
Tobacco chewing	1	3.3%	14	46.7%	15	50%	>0.05
Tobacco smoking	0	-	8	26.7%	8	26.7%	
Smoking and chewing tobacco	0	-	1	3.3%	1	3.3%	
No smoking/chewing tobacco	1	3.3%	5	16.7%	6	20%	
Total	2	6.7%	28	93.3%	30	100%	

N= Number of patients having EGFR mutation. SD= Standard Deviation. p Value= Probability Value.

**Table 4 t04:** Association of EGFR Mutation and TNM Stage of tumor

TNM Stage	EGFR Mutation				Total		p Value
	Present		Absent				
	N	%	N	%	N	%	
0	0	-	1	3.3%	1	3.3%	>0.05
1	0	-	11	36.7%	11	36.7%	
2	1	3.3%	3	10%	4	13.3%	
3	0	-	3	10%	3	10%	
4	1	3.3%	10	33.4%	11	36.7%	
Total	2	6.7%	28	93.3%	30	100%	

N= Number of patients having EGFR mutation. SD= Standard Deviation. p Value= Probability Value.

**Table 5:** Association of EGFR Mutation and Grade of tumor

Grade	EGFR Mutation				Total		p Value
	Present		Absent				
	N	%	N	%	N	%	
1	2	6.7%	21	70%	23	76.7%	>0.05
2	0	-	5	16.7%	5	16.7%	
3	0	-	2	6.7%	2	6.7%	
Total	2	6.7%	28	93.3%	30	100%	

N= Number of patients having EGFR mutation. SD= Standard Deviation. p Value= Probability Value.

## DISCUSSION

*EGFR* became one of the commonly investigated targets for new therapies in solid organ tumors. However, limited studies have been done on these mutations in cases of HNSCC globally.

In 2017, Christos Perisanidis^9^ undertook a systematic review of *EGFR* mutations in HNSCC, comparing the statistics of *EGFR* mutations worldwide. The *EGFR* polymorphic variation in HNSCC was found to be specific to geographic regions and ethnicity with varying prevalence in different populations. Among the four Continents, Southeast Asia (4.9%), followed by North America (2.7%), showed the highest prevalence. When considering individual countries, the highest prevalence of 15.1% was found in the Republic of Korea, followed by the Czech Republic (6.9%). In our study, the incidence of *EGFR* mutations was 6.6% (2/30 cases), which is comparable with the study by Smilek et al.^10^ 6.9% (2/29 cases). Our study found that both mutations were found in tumors arising from the tongue. This is consistent with other studies by Smilek et al.^10^, Nagalakshmi et al.,^11^ and the systemic review by Perisanidis et al.^9^.

Our study failed to show any correlation of the presence of these mutations with age, gender, smoking, stage, or grade of the tumor, which is consistent with the prospective clinical study of *EGFR* mutations by Smilek et al.^10^ The Smilek study comprised 29 cases of HNSCC and detected two cases (stage III and IV) with the *EGFR* mutation. The *EGFR* mutation with a deletion in exon 19 was associated with a worse prognosis, presence of recurrence, and no response to treatment.

Nagalakshmi et al.,^11^ in a hospital-based case-control study, reported a significant association of *EGFR* mutations with an advanced stage of HNSCC, history of tobacco/alcohol, and age more than 49 years. Bahassi et al.^12^ reported a case with mutated *EFGR,* which presented a positive impact on treatment.

The comparison of the results of our study with other studies is depicted in Table 6.

**Table 6:** Comparison of our study results with two other studies on EGFR mutation in HNSCC

Parameters		Current Study	Smilek et al.^10^	Nakalakshmi et al.^11^
Total cases		30	29	129
Males		25	24	65
Females		05	05	64
Age	> or=50 years	21	15	89
	< 50 years	9	14	40
Country		India	Czech Republic	India
Tumor site	Oral cavity	26	02	36
	Oropharynx	01	19	16
	Hypopharynx	0	05	00
	Larynx	03	03	06
TNM Stage	Stage I	11	0	7
	Stage II	04	0	9
	Stage III	03	05	17
	Stage IV	12	24	32
Tumor Grade	Grade 1	23	03	40
	Grade 2	05	17	17
	Grade 3	02	09	08
EGFR mutation		02/30 6%	02/29 6%	81.39%
Samples		FTT	FTT	FTT
Method		PCR	PCR	PCR and SSCP
Mutation		p.S768I, p.T790M on Exon 20	Exon 19 del	Exon 18, 19, 20 mutations

FTT= fresh tumor tissue; SSCP= single strand confirmatory polymorphism

The variation in *EGFR* mutations results may be attributed to various detection methods used to study these mutations, which present varied sensitivity and specificity.

In the study by Nagalakshmi et al.,^11^ the single strand confirmatory polymorphism was the molecular technique used along with Sanger Sequencing, hence enabling detection of a large number of mutations. The study by Smilek et al.^10^ and the present study, on the other hand, were done to study specific mutations that have a role in targeted therapy using primer and probes by PCR technology.

## CONCLUSION

HNSCC is one of the commonest malignancies in India and has an aggressive course. Mutations in the tyrosine kinase domain of the *EGFR* gene on Exons 19 to 21 is one of the multiple molecular pathways of tumorigenesis in head and neck squamous cell carcinoma. Studies have shown that *EGFR TK* mutations are present in a subset of these tumors. TKI therapy may have a role in the treatment of patients harboring these mutations. However, the challenge is finding out the specific *EGFR* mutations related to resistance or response to anti-EGFR or other targeted therapies. Larger studies and clinical trials may help provide targeted therapy to a subset of patients found with *EGFR TKI* mutations.
